# In Vivo Assessment of VCAM-1 Expression by SPECT/CT Imaging in Mice Models of Human Triple Negative Breast Cancer

**DOI:** 10.3390/cancers11071039

**Published:** 2019-07-23

**Authors:** Christopher Montemagno, Laurent Dumas, Pierre Cavaillès, Mitra Ahmadi, Sandrine Bacot, Marlène Debiossat, Audrey Soubies, Loic Djaïleb, Julien Leenhardt, Nicolas De Leiris, Maeva Dufies, Gilles Pagès, Sophie Hernot, Nick Devoogdt, Pascale Perret, Laurent Riou, Daniel Fagret, Catherine Ghezzi, Alexis Broisat

**Affiliations:** 1 Laboratory of Bioclinical Radiopharmaceutics, Universite Grenoble Alpes, Inserm, CHU Grenoble Alpes, LRB, 38000 Grenoble, France; 2 Advanced Accelator Applications, 01630 Saint-Genis-Pouilly, France; 3 Natural Barriers and Infectiosity, Universite Grenoble Alpes, CNRS, CHU Grenoble Alpes, TIMC-IMAG, 38000 Grenoble, France; 4 Biomedical Department, Centre Scientifique de Monaco, 980000 Monaco, Monaco; 5 Institute for Research on Cancer and Aging of Nice, Universite Cote d’Azur, CNRS UMR 7284, INSERM U1081, Centre Antoine Lacassagne, 061489 Nice, France; 6 Laboratory of In Vivo Cellular and Molecular Imaging, ICMI-BEFY, Vrije Universiteit Brussel, Laarbeeklan 103, B-1090 Brussels, Belgium

**Keywords:** triple negative breast cancer, VCAM-1, SPECT imaging, sdAbs

## Abstract

Recent progress in breast cancer research has led to the identification of Vascular Cell Adhesion Molecule-1 (VCAM-1) as a key actor of metastatic colonization. VCAM-1 promotes lung-metastases and is associated with clinical early recurrence and poor outcome in triple negative breast cancer (TNBC). Our objective was to perform the in vivo imaging of VCAM-1 in mice models of TNBC. The Cancer Genomic Atlas (TCGA) database was analyzed to evaluate the prognostic role of VCAM-1 in TNBC. MDA-MB-231 (VCAM-1+) and control HCC70 (VCAM-1-) TNBC cells were subcutaneously xenografted in mice and VCAM-1 expression was assessed in vivo by single-photon emission computed tomography (SPECT) imaging using ^99m^Tc-cAbVCAM1-5. Then, MDA-MB-231 cells were intravenously injected in mice and VCAM-1 expression in lung metastasis was assessed by SPECT imaging after 8 weeks. TCGA analysis showed that VCAM-1 is associated with a poor prognosis in TNBC patients. In subcutaneous tumor models, ^99m^Tc-cAbVCAM1-5 uptake was 2-fold higher in MDA-MB-231 than in HCC70 (*p* < 0.01), and 4-fold higher than that of the irrelevant control (*p* < 0.01). Moreover, ^99m^Tc-cAbVCAM1-5 uptake in MDA-MB-231 lung metastases was also higher than that of ^99m^Tc-Ctl (*p* < 0.05). ^99m^Tc-cAbVCAM1-5 is therefore a suitable tool to evaluate the role of VCAM-1 as a marker of tumor aggressiveness of TNBC.

## 1. Introduction

Breast cancer (BC) is the most common female malignancy, accounting for more than 30% of all malignant tumors in women [1]. Breast cancer is a heterogeneous disease consisting of various subtypes with distinct molecular and pathological profiles [2,3]. Triple Negative Breast Cancer (TNBC) subtypes represent 10% to 20% of BC and are characterized by the lack of progesterone receptor, estrogen receptor and human epidermal growth factor receptor 2 expression [4]. TNBC are mostly associated with poor clinical outcome and high rate of metastasis and relapse following treatment [5,6]. Lung, bones, brain and liver are the most common sites of distant metastasis. Despite intense clinical research efforts, only limited advances have been obtained in the management of BC metastases.

Recent studies have led to the identification of new genes and mechanisms that mediate metastatic colonization [7]. Among them, Vascular Cell Adhesion Molecule-1 (VCAM-1) plays a key role in BC progression and metastatic processes [8,9]. VCAM-1 belongs to the immunoglobulin super family group of adhesion molecules. It is a 110 kDa glycoprotein mainly expressed at the endothelial cells surface during inflammation, but also by macrophages and dendritic cells [10]. The ability of VCAM-1 expressed by endothelial cells to bind tumor cells suggest that it could contribute to metastatic spread. Indeed, VCAM-1 expression on endothelial cells plays a key role in angiogenesis, in tumor cell transmigration, and therefore promotes tumor development and dissemination of tumor cells [11].

In the last few years, a growing interest into VCAM-1 expression on tumor cells has emerged. In BC, the overexpression of VCAM-1 on tumor cells correlates with early relapse and poor patient outcome [12,13]. The direct interaction of VCAM-1 with its ligand Very-Late Antigen -4 (VLA-4) expressed on leukocytes allows tumor cell survival in lungs and consequently lung metastasis [14]. Moreover, VCAM-1 is involved in the transition from dormant micro-metastases to overt macro-metastases in bones, a turning point in BC progression [13,15]. Inhibition of the VCAM-1 and VLA-4 interaction impairs bone metastasis progression and lung colonization [13,14,16]. VCAM-1 is also involved into chemoresistance and tumor immune escape [17,18,19]. Therefore, VCAM-1 is a key player with multiple functionalities in directing the metastatic spread. VCAM-1 expression could be induced by pro-inflammatory cytokines, such as Tumor Necrosis Factor-α (TNF-α), IL-1 or IL-6, major mediators of tumor progression [19].

To assess the role of VCAM-1 in the metastatic phenotype of BC, nuclear imaging may represent a powerful tool. We recently developed a single domain antibody (sdAb)-based radiotracer targeting VCAM-1 called ^99m^Technetium-cAbVCAM1-5 (^99m^Tc-cAbVCAM1-5), which is ongoing clinical transfer for detection of inflamed atherosclerosis lesions [20,21]. The aim of the present study was to assess VCAM-1 expression using ^99m^Tc-cAbVCAM1-5 in subcutaneous and lung metastasis mice models of TNBC.

## 2. Results

### 2.1. VCAM-1 Is Overexpressed in TNBC and Associated with Decreased OS

**Figure 1:**
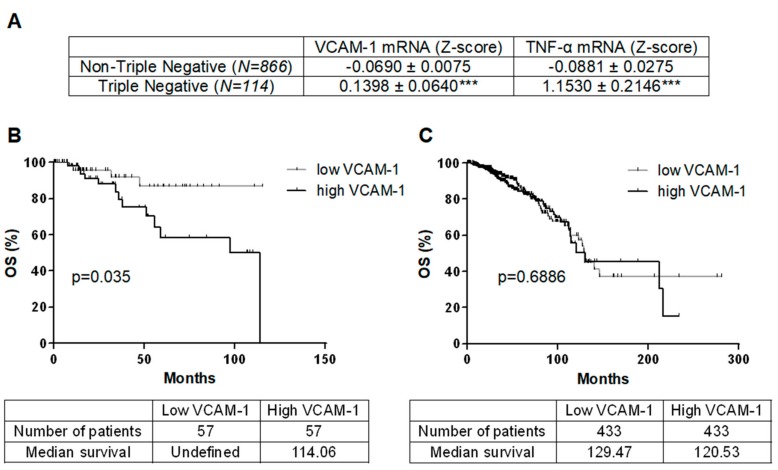
VCAM-1 is overexpressed in Triple-Negative Breast Cancer (TNBC) and associated with a decrease of overall survival. (**A**) Expression of VCAM-1 and TNF-α in TNBC and Non-TNBC were compared in TCGA datasets for breast cancer. Values represent the z scores for log_2_-transformed normalized RNA-seq read counts. (**B**,**C**) Kaplan-Meier analysis of OS of TNBC (**B**) and Non-TNBC (**C**) patients. OS (months) were calculated from patient subgroups with VCAM-1 mRNA levels that were less or greater than the median value. Statistical significance (*p*-value) is indicated. *** *p* < 0.001. Nb: Number.

980 breast cancer patients of The Cancer Genomic Atlas (TCGA) were analyzed for VCAM-1 mRNA levels and subsequent overall survival (OS) follow-up (Figure 1). VCAM-1 expression was higher in TNBC (*n* = 114) as compared to non-TNBC patients (*n* = 866) (*p* < 0.001, Figure 1A). High VCAM-1 expression was associated with decreased OS in TNBC but not in non-TNBC patients (respectively *p* = 0.035 and *p* = 0.6886, Figure 1B,C). TNF-α mRNA expression is overexpressed in TNBC as compared to non-TNBC patients (*p* < 0.001, Figure 1A).

### 2.2. MDA-MB-231 Cells Overexpress VCAM-1 mRNA and Protein

**Figure 2:**
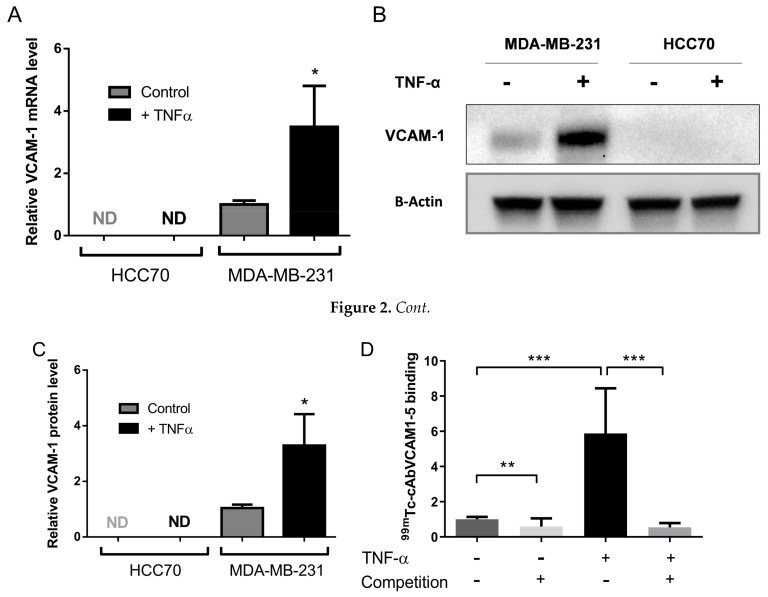
MDA-MB-231 cells overexpress VCAM-1 mRNA and protein upon TNF-α stimulation. MDA-MB-231 and HCC70 cells were treated or not for 18 h with TNF-α (50 ng/mL) and VCAM-1 expression was assessed by RT-qPCR (**A**) and WB (**B**). (**C**) Bands of WB were quantified using ImageJ software. (**D**) ^99m^Tc-AbVCAM1-5 binding was performed on MDA-MB-231 cells stimulated or not with TNF-α, in the presence or absence of a 100-fold excess of unlabeled cAbVCAM1-5. Unspecific binding was estimated by ^99m^Tc-AbVCAM1-5 incubation on HCC70 cells. Results are expressed in fold-change vs. untreated and unstimulated MDA-MB-231 *N* = 6 per condition. * *p* < 0.05; ** *p* < 0.01; *** *p* < 0.001; ND, not detected.

The expression of VCAM-1 was first assessed on two TNBC cell lines. VCAM-1 mRNA levels were undetectable at the basal level or following TNF-α stimulation in HCC70 cells. However, MDA-MB-231 cells expressed VCAM-1 mRNA basal levels. They were induced by a 3.5-fold upon TNF-α stimulation (1.0 ± 0.1 vs. 3.5 ± 1.3, *p* < 0.05, Figure 2A). Consistently, VCAM-1 protein was undetectable in HCC70 cells whereas MDA-MB-231 expressed basal VCAM-1 protein levels and TNF-α induced a 3.5-fold increase of these basal levels (Figure 2B,C, Figure S1).

### 2.3. ^99m^Tc-cAbVCAM1-5 Binds Specifically to VCAM-1 Expressing TNBC Cells

Unlabeled cAbVCAM1-5 bound to VCAM-1-positive MDA-MB-231 but not to VCAM-1-negative HCC70 cells (Figure S2). ^99m^Tc-cAbVCAM1-5 affinity was evaluated on both mouse and human recombinant protein and found to be in the nanomolar range scale (Kd = 12.5 ± 3.6 nM for mVCAM-1 and 36.3 ± 7.0 nM for hVCAM-1) (Figure S3). To further demonstrate the specificity of the binding on MDA-MB-231 cells, in vitro competition studies were then performed to determine the ability of the radiolabeled ^99m^Tc-cAbVCAM1-5 to bind specifically to MDA-MB-231 (Figure 2D). ^99m^Tc-cAbVCAM1-5 binding to TNF- α stimulated cells was 5-fold higher than to unstimulated cells (5.8 ± 2.6 vs. 1.0 ± 0.2, respectively, *p* < 0.001). Moreover, the addition of an excess of unlabeled cAbVCAM1-5 significantly decreased ^99m^Tc-cAbVCAM1-5 binding on unstimulated and on stimulated MDA-MB-231 cells by 42% (*p* < 0.01) and 90% (*p* < 0.001), respectively. These results demonstrated that ^99m^Tc-cAbVCAM1-5 binds specifically to VCAM-1 expressing cells.

### 2.4. SPECT/CT Imaging of VCAM-1 in Subcutaneous Tumor Model

**Figure 3:**
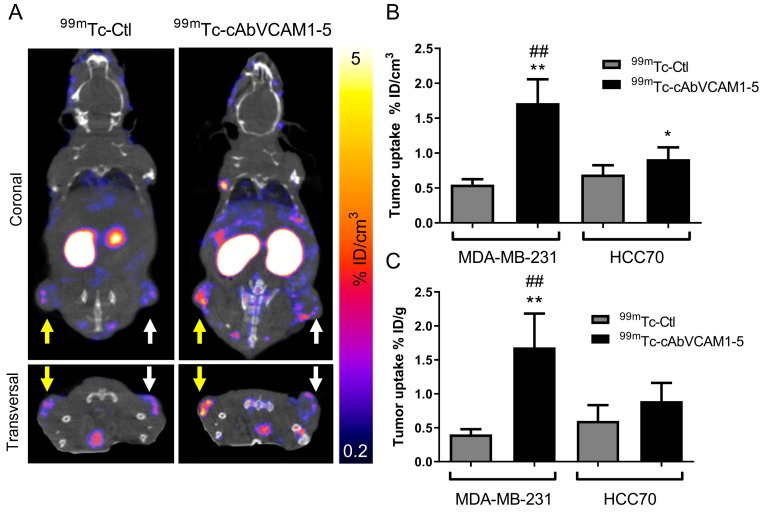
In vivo biodistribution of ^99m^Tc-cAbVCAM1-5 in mice bearing HCC70 and MDA-MB-231 tumor xenografts. (**A**) Representative coronal and transversal views of fused SPECT/CT images of HCC70 (right hind limb, white arrow) and MDA-MB-231 (left hind limb, yellow arrow) tumor-bearing mice at 1 h after i.v injection of ^99m^Tc-cAbVCA1M-5 or ^99m^Tc-Ctl. (**B**) In vivo quantification of ^99m^Tc-cAbVCAM1-5 and ^99m^Tc-Ctl tumor uptake from SPECT images. (**C**) Ex vivo quantification of ^99m^Tc-cAbVCAM1-5 and ^99m^Tc-Ctl tumor uptake from post-mortem biodistribution studies. Results were expressed as % ID/cm^3^ (imaging) and % ID/g (ex vivo) of tumor. Statistics: * *p* < 0.05, ** *p* < 0.01 vs. ^99m^Tc-Ctl; *## p* < 0.01 vs. HCC70.

^99m^Tc-cAbVCAM1-5 potential to perform in vivo imaging of VCAM-1-expressing tumors was then evaluated. Whole-body SPECT/CT images of mice subcutaneously implanted with MDA-MB-231 and HCC70 tumors are presented in Figure 3A and in Figure S4. ^99m^Tc-cAbVCAM1-5 uptake was readily detectable in MDA-MB-231 tumors, whereas a weak signal was detectable from HCC70 tumor and from MDA-MB-231 or HCC70 tumors using the irrelevant ^99m^Tc-Ctl (Figure 3A). Quantification of SPECT images confirmed that ^99m^Tc-cAbVCAM1-5 uptake in MDA-MB-231 tumors (1.7 ± 0.5% ID/cm^3^) was significantly higher than in HCC70 tumors (0.9 ± 0.2% ID/cm^3^, *p* < 0.01), whereas ^99m^Tc-Ctl uptake was similar in HCC70 and in MDA-MB-231 tumors (0.6 ± 0.1 vs. 0.6 ± 0.2% ID/cm^3^, respectively) (Figure 3B) and significantly lower than ^99m^Tc-cAbVCAM1-5 tumor uptake in MDA-MB-231 (*p* < 0.01) and HCC70 tumors (*p* < 0.05). The biodistribution of ^99m^Tc-cAbVCAM1-5 and ^99m^Tc-Ctl determined ex vivo by gamma-well counting was in accordance with that obtained following SPECT image quantification, thereby demonstrating the accuracy of the method (Figure 3C and Figure S5). Specific ^99m^Tc-cAbVCAM1-5 uptake in lymphoid organs was observed which was consistent with VCAM-1 expression (Table S1). 

### 2.5. Ex Vivo Assessment of VCAM-1 Expression in Subcutaneous Tumors

**Figure 4:**
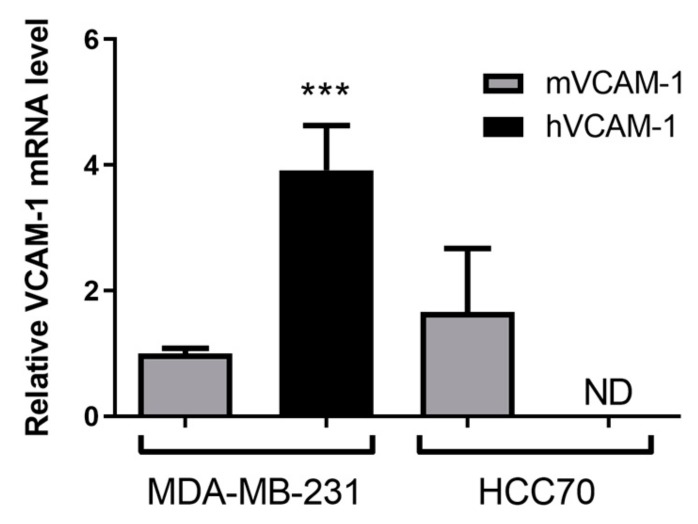
Ex vivo assessment of VCAM-1 expression by RT-qPCR. 50 mg of MDA-MB-231 and HCC70 xenografts were harvested and tested for human and mice VCAM-1 expression by RT-qPCR. *** *p* < 0.001 vs. MDA-MB-231 mVCAM-1.

Since VCAM-1 is also involved in inflammatory and angiogenic processes, we next determined which part of its expression was attributable to tumor cells (human VCAM-1), or to the inflammatory and endothelial cells (mouse VCAM-1) using RT-qPCR (Figure 4). Consistently with in vitro results, hVCAM-1 mRNA was expressed by MDA-MD-231 but was not detectable in HCC70 tumors. Moreover, hVCAM-1 expression was nearly 4-fold higher than that of mVCAM-1 in MDA-MB-231 xenograft tumors (*p* < 0.001), suggesting that the hVCAM-1 expression by tumor cells was the predominant form present in MDA-MB-231 subcutaneous xenografts. Interestingly, no significant difference was observed in mVCAM-1 mRNA expression between HCC70 and MDA-MB-231 tumors (1.6 ± 1.0 vs. 1.0 ± 0.1). This results strongly suggests that the signal obtained on SPECT/CT images reflects VCAM-1-expressing tumor cells. 

### 2.6. SPECT/CT Imaging of VCAM-1 in an Experimental Metastasis Model

**Figure 5:**
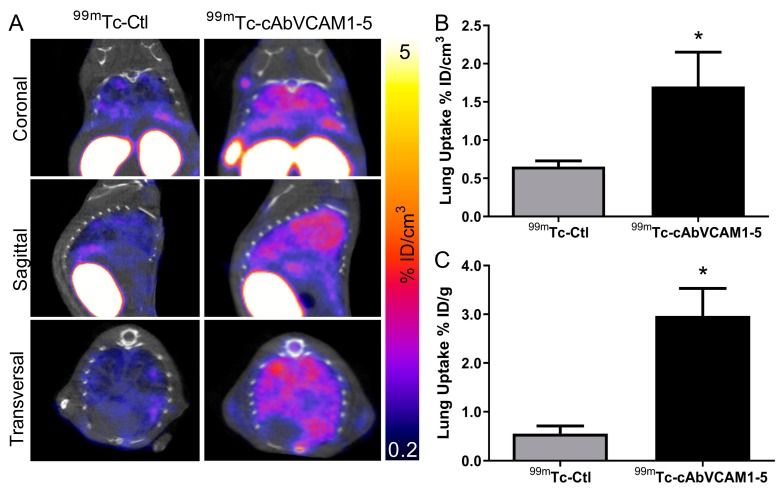
In vivo biodistribution of ^99m^Tc-cAbVCAM1-5 in a pulmonary experimental metastasis model. (**A**) Representative coronal and transversal views of fused SPECT/CT images of MDA-MB-231 lung metastases at 1 h after i.v. injection of ^99m^Tc-cAbVCAM1-5 or ^99m^Tc-Ctl. (**B**) In vivo quantification of ^99m^Tc-cAbVCAM1-5 and ^99m^Tc-Ctl lung uptake from SPECT images. (**C**) Ex vivo quantification of ^99m^Tc-cAbVCAM1-5 and ^99m^Tc-Ctl lung uptake from post-mortem biodistribution studies. Results were expressed as % ID/cm^3^ (imaging) and % ID/g (ex vivo) of tumor. Statistics: * *p* < 0.05 vs. ^99m^Tc-Ctl.

Because VCAM-1 is involved in lung colonization we next investigated the ability of ^99m^Tc-cAbVCAM1-5 to perform its imaging in an experimental metastasis model. Representative SPECT/CT images are presented in Figure 5A and in Figure S6.

**Figure 6:**
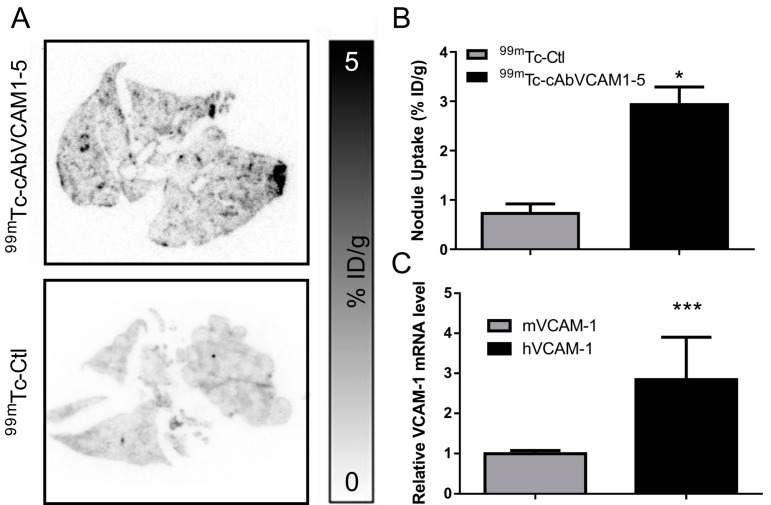
Ex vivo assessment of ^99m^Tc-cAbVCAM1-5 uptake in metastatic nodules and assessment of VCAM-1 expression by RT-qPCR. (**A**) Autoradiography on 20 µm metastasis-containing lung slices. (**B**) Autoradiographic image quantification of ^99m^Tc-cAbVCAM1-5 and ^99m^Tc-Ctl uptake in MDA-MB-231 metastatic nodules. * *p* < 0.05 vs. ^99m^Tc-Ctl. (**C**) 50 mg of lung containing metastases (MDA-MB-231) were harvested and tested for human and mice VCAM-1 expression by RT-qPCR. *** *p* < 0.001 vs. MDA-MB-231 mVCAM-1.

^99m^Tc-cAbVCAM1-5 lung uptake was readily observable, whereas a weak signal was obtained with ^99m^Tc-Ctl (Figure 5A). Quantification of SPECT/CT images confirmed that the lung uptake was 2.5-fold higher with ^99m^Tc-cAbVCAM1-5 than ^99m^Tc-Ctl (1.7 ± 0.4 vs. 0.7 ± 0.1% ID/cm^3^, *p* < 0.05, Figure 5B). When performing image quantification of pulmonary activity, the volume of interest contains a mixture of tissue and air, thereby leading to an underestimation of the uptake. ^99m^Tc-cAbVCAM1-5 lung uptake was therefore further evaluated using ex vivo gamma-well counting (Figure 5C) and autoradiography (Figure 6A,B). Using these two technics, ^99m^Tc-cAbVCAM1-5 uptake was found to represent ~3% ID/g and 4–5 fold-higher value than that obtained with ^99m^Tc- Ctl (*p* < 0.05 for both technics). ^99m^Tc-cAbVCAM1-5 uptake was also evaluated in lung-metastasis free mice and found to be 3-fold lower than lung-metastasis bearing mice (*p* < 0.05, Figure S7).

### 2.7. Ex Vivo Assessment of VCAM-1 Expression in the Experimental Metastasis Model

As in the subcutaneous xenograft study, VCAM-1 lung expression was assessed by RT-qPCR (Figure 6C). Both mVCAM-1 and hVCAM-1 mRNA were expressed, with the predominant expression of hVCAM-1 in tumors. Indeed, hVCAM-1 was nearly 3-fold more expressed than mVCAM-1 (2.8 ± 1.1 vs. 1.0 ± 0.1, *p* < 0.001). This results strongly suggests that the ^99m^Tc-cAbVCAM1-5 imaging reflects VCAM-1 tumor cells expression in this model.

## 3. Discussion

Despite major advances in fundamental knowledge and therapeutic opportunities, BC remains the leading form of malignancy among women [1]. If the 5-year relative survival of BC is almost 100% when the cancer is restricted to the breast, the prognosis of patients with metastatic BC is unfavorable with a 5-year survival rate of 25% [22]. Moreover, among women initially diagnosed without metastasis, 20 to 25% will develop a metastatic disease in the next 5 years [23]. Despite intense clinical research efforts, there is still a strong need for novel molecular target and therapies to improve management of BC metastases [24]. The identification of genes and mechanisms involved in metastatic processes and the development of effective treatments against metastatic BC are outstanding challenges in current experimental and clinical research. In the past few years, growing interest into tumorigenicity and metastatic processes has led to the identification of VCAM-1 as a key actor for tumor growth, metastasis and angiogenesis [25].

In BC, VCAM-1 expression on tumor cells is an important actor for metastatic colonization of lungs and bones [9]. In the lungs VCAM-1 binds to α4β1 integrin expressed on macrophages triggering the activation of the PI3K/Akt survival pathway in cancer cells [14]. In bone, VCAM-1 expressing tumor cells binds to α4β1 integrin-expressing osteoclast progenitors to mediate osteolytic metastasis [13]. Moreover, VCAM-1 expression seems to be correlated to poor outcome in BC [12]. The results provided by the TCGA analysis showed that VCAM-1 is overexpressed in TNBC in comparison to Non-TNBC. Moreover, VCAM-1 expression is associated with a poor prognosis only in TNBC patients. In addition to BC, VCAM-1 increased expression has also been described in other cancer types such as glioblastoma, gastric and ovarian cancer. In these cancers, VCAM-1 expression correlated with the tumor grade [26,27,28]. Considering the role of VCAM-1 in directing the metastatic spread, VCAM-1 imaging agents could be used to (1) understand the biological role of VCAM-1 in metastatic processes and (2) to evaluate the prognostic value of VCAM-1 expression in clinical practice. Therefore, radiotracers have been developed for VCAM-1 imaging in tumors [29,30].

Our group recently developed ^99m^Tc-cAbVCAM1-5, a radiotracer targeting VCAM-1 which is ongoing clinical transfer for the detection of inflamed atherosclerotic lesions [20,21,31]. The purpose of the present study was to evaluate this new tool for the pre-clinical nuclear imaging of VCAM-1 in TNBC. Two TNBC cell lines were employed, MDA-MB-231 and HCC70. The MDA-MB-231 cell line is highly metastatic whereas few results were available on HCC70. In vitro experiments demonstrated that MDA-MB-231 expressed hVCAM-1 mRNA and protein, and that expression was increased after TNF-α stimulation which is consistent with previous studies [19]. TNF-α is a key activator of VCAM-1 through NF-κB signaling [32]. A bundle of evidences links TNF-α and NF-κB pathway to tumor survival, growth and invasion [33,34]. The results provided by the TCGA showed higher TNF-α expression in TNBC in comparison to Non-TNBC tumors. These results suggest that the TNF-α/VCAM-1 axis is a relevant target in TNBC. As demonstrated by in vitro competition studies, the ^99m^Tc-cAbVCAM1-5 tracer specifically binds human VCAM-1. Using this imaging agent, subcutaneous MDA-MB-231 tumors were successfully visualized by SPECT/CT imaging, whereas significantly lower signals were found in HCC70 tumors (which do not express hVCAM-1 mRNA and protein) or using the negative irrelevant control sdAb, thereby indicating that the signal was specific and predominantly attributable to tumor cell expressing VCAM-1 rather than murine endothelial or inflammatory cells. These results were in agreement with the level of murine and human VCAM-1 mRNA determined in tumor biopsies by RT-qPCR. Additional studies are however warranted in order to investigate if, similarly to that has been observed in vitro, in vivo hVCAM-1 expression in MDA-MB-231 tumor cells can be increased by TNF-α or other cytokines present in the microenvironment, leading to increased ^99m^Tc-cAbVCAM1-5 uptake.

Therefore, ^99m^Tc-cAbVCAM1-5 is a validated tool to investigate the role of VCAM-1 in metastatic processes. However our results support that using VCAM-1 as a tumor inflammatory marker should be carefully considered due to the potential expression of VCAM-1 by tumor cells themselves.

Because of VCAM-1 is aberrantly expressed in BC cells and mediates lung metastasis, ^99m^Tc-cAbVCAM1-5 imaging was further studied in an experimental lung metastasis model with MDA-MB-231 cells. According to SPECT/CT quantifications, ^99m^Tc-cAbVCAM1-5 lung uptake was 3-fold higher than that of ^99m^Tc-Ctl whereas it was found to be 5-fold higher by ex vivo gamma-well counting. This difference can be attributed to the fact that when performing in vivo lung imaging quantification, the volume of interest contains a mixture of tissue and air, thereby leading to underestimation of the uptake.

Autoradiography further confirmed that ^99m^Tc-cAbVCAM1-5 uptake was localized in metastatic nodules, and RT-qPCR showed that, in the whole lung, hVCAM-1 expression was found to be 3-fold higher than mVCAM-1 indicating that ^99m^Tc-cAbVCAM1-5 uptake highlights presence of tumor cells rather than inflammatory processes. This result is consistent with a previous study demonstrating VCAM-1 tumor expression rather than endothelial cells one in lung metastasis of BC patients [14]. ^99m^Tc-cAbVCAM-1 is therefore a validate tool to study the prognostic value of VCAM-1 in the metastatic disease.

Other VCAM-1-targeting radiotracers have been developed. ^99m^Tc-cAbVCAM1-5 tumor uptake was comparable to that obtained by Scalici et al. using ^111^In-tVCAM-4, an indium-111 labeled peptide targeting VCAM-1 [30]. Indeed, in an experimental mouse model of metastatic ovarian cancer, ^111^In-tVCAM-4 uptake in tumor was of ~2% ID/g at 4 h post-injection. Using a ^68^Ga-labeled Single Chain Variable Fragment, Zhang et al. obtained a ~5% ID/g tumor uptake in a mouse model of melanoma. Nevertheless, due to the fast blood clearance of sdAbs-based imaging agents, ^99m^Tc-cAbVCAM1-5 tumor-to-blood ratio (5 at 2 h) favorably compared to that of this imaging agent (2 at 3 h) [29].

## 4. Materials and Methods

### 4.1. Patients—Online Data

Normalized RNA sequencing (RNA-Seq) data of The Cancer Genome Atlas (TCGA) were downloaded from cBioportal (Breast Invasive Carcinoma—TCGA Provisional; RNA-Seq V2) [35]. Data were available for 980 BC tumor samples (866 Non-TNBC and 114 TNBC). The results published here are based upon data generated by the TCGA Research Network [36,37].

### 4.2. Cell Lines and Culture Conditions

MDA-MB-231 cells were cultured with Dulbecco’s Modified Eagle’s Medium supplemented with 10% fetal bovine serum and 1% penicillin-streptomycin. HCC70 cells were cultured using Roswell Park Memorial Institute-1640 medium, supplemented with 10% fetal bovine serum and 1% penicillin-streptomycin.

### 4.3. RT-qPCR Assay

HCC70 and MDA-MB-231 cells were stimulated or not with Tumor Necrosis Factor-α (TNF-α, 50 ng/mL, Peprotech^®^, Rocky Hill, NJ, USA) for 18 h. Cell lysis and extraction of total RNA from cell lines were performed using PureLink^TM^ RNA Mini Kit (ThermoFisher Scientific, Illkirch, France) according to the manufacturer’s protocol. Reverse transcription (RT) was performed using an iScript Reverse transcription kit (iScript RT supermix; BioRad, Hercules, CA, USA). The reaction mixtures were incubated at 25 °C for 5 min, 46 °C for 20 min, 95 °C for 1 min and held at 4 °C. For the assessment of VCAM-1 expression in xenograft tumors and in metastatic nodules, 20–50 mg of tissue was ground (PureLink^TM^ RNA Mini Kit; ThermoFisher Scientific), and the same protocol as for the in vitro assay was applied. Quantitative Polymerase Chain Reaction (qPCR) was performed on the resulting cDNA with the Fast SYBR Green Master Mix (ThermoFisher Scientific, Illkirch, France), using a Real Time PCR system (Applied Biosystem StepOne Plus; ThermoFisher Scientific). The primer sequences were as follows: human VCAM-1 (hVCAM-1) Forward, 5′-AGTTGAAGGATGCGGGAGTA-3′, Reverse 5′-ACCCCTTCAT GTTGGCTTTTC-3′; murine VCAM-1 (mVCAM-1) Forward, 5′- GCCACCCTCACCTTAATTGC -3′, Reverse 5′- TCAGAACAACCGAATCCCCA-3’. The expression levels of mRNAs were normalized to the endogenous control actin, and were calculated with the formula 2^−ΔΔCT^. The primer sequence for actin was as follows: Forward, 5′-CTCCTGAGCGCAAGTACTCC-3′, Reverse 5′-TGTTTTCT GCGCAAGTTAGG-3′.

### 4.4. Western Blot Assay

HCC70 and MDA-MB-231 cells were treated or not with TNF-α (50 ng/mL) for 18 h. Total proteins were extracted with RIPA buffer. Proteins were separated by 7.5% denaturing SDS-PAGE and transferred onto a nitrocellulose membrane. The membrane was incubated with the anti-VCAM-1 antibody (1/2000; Rabbit anti-VCAM-1, ab134047 Abcam®, Cambridge, UK). As a loading control, the membrane was probed with an anti-β-actin antibody (1/10,000; Beckton Dickinson). Revelation was assessed using the chemiluminescence ECL kit (BioRad). Bands were quantified by densitometry using ImageJ software.

### 4.5. Flow Cytometry

MDA-MB-231 and HCC70 cells (200.000) treated or not with TNF-α (50 ng/mL) for 18 h were collected and washed with phosphate buffered saline solution (PBS). Cells were incubated with cAbVCAM1-5 (10 μg/mL) and then with an anti-poly histidine antibody (anti-6x His tag antibody, ab9108 Abcam), followed by a AlexaFluor®488-anti-Rabbit-IgG (Goat anti-Rabbit IgG H&L, AlexaFluor® 488, ab150077 Abcam). Binding was measured on a FACS Accuri C6 analyzer (BD Biosciences, San Jose, CA, USA). Background fluorescence was determined by measuring the fluorescent signal from cells stained with the irrelevant control sdAb.

### 4.6. Saturation Binding Experiments and In Vitro Competition Studies

Saturation binding experiments was determined on 96-well plates coated with mouse or human recombinant VCAM-1 protein (100 ng/well; R&D Systems, Minneapolis, MN, USA). Serial diluations of ^99m^Tc-cAbVCAM1-5 were incubated for 1 h at room temperature before being washed 5 times with PBS-polysorbate 0.1%. The radioactivity in each well was then determined using a γ-counter (Wizard^2^; Perkin Elmer, Courtaboeuf, France) and corrected for unspecific binding. Binding curves were fitted using a non linear regression equation (specific binding: *y* = B_max_ × *x*/(K_D_ + *x*), with *x* being the radioligand concentration, K_D_ being the dissociation constant, and B_max_ being the maximum number of binding sites, or receptor density) (GraphPad Prism, version 6, software, San Diego, CA, USA), to determine K_D_ values.

For in vitro competition studies, 12,000 HCC70 and MDA-MB-231 cells were coated in 96-well plates (Stripwell^TM^ Plate, Corning^®^, Corning, NY, USA) in their respective culture medium. After 24 h, cells were treated with 50 ng/mL of TNF-α for 18 h. Then, cells were rinsed with PBS and fixed with paraformaldehyde (PFA) 4% for 10 min. Following 1 h of saturation with PBS-BSA 1%, cells were incubated with 30 nM of ^99m^Tc-cAbVCAM1-5 in the absence or presence of a 100-fold excess of unlabeled cAbVCAM1-5 at room temperature for 1 h before being washed 5 times with PBS-Tween 0.05%. Bound activity was determined using a gamma-counter (Wizard^2^, Perkin Elmer) and results were corrected from nonspecific binding determined on HCC70 cells.

### 4.7. Tumor Models

All animal procedures conformed to French government guidelines (Articles R214-87 to R214-126; European directive 2010/63/UE). They were performed in an approved facility (C385161 0005) under permit APAFIS#3690-2016011916045217 v4 and APAFIS#8683-2017012611031820 v3 from the French Ministry of Research. To evaluate ^99m^Tc-cAbVCAM1-5 biodistribution and tumor uptake, 12 female BALB/c nu/nu mice (5 weeks old) were subcutaneously inoculated into the left flank with MDA-MB-231 (3 × 10^6^) and into the right flank with HCC70 (3 × 10^6^), in a 2:1 PBS/Matrigel^®^ (Corning) mixture. The tumors were allowed to grow for 3 weeks to reach ~150–200 mm^3^. For the experimental metastasis model, 9 female C.B17 SCID (Severe Combined ImmunoDeficient) mice (5 weeks old) were intravenously injected with MDA-MB-231 cells (1 M in 150 µL) in PBS. Eight weeks later, SPECT/CT imaging and biodistribution studies were performed.

### 4.8. SPECT/CT Imaging

For the subcutaneous xenograft study, mice were divided in 2 groups: tumor bearing mice injected with (i) the human/mouse cross-reactive sdAb ^99m^Tc-cAbVCAM1-5 (*n* = 6) or (ii) with the irrelevant control sdAb, ^99m^Tc-Ctl (*n* = 6). The previously described BcII10a control sdAb was used in this study (20). SPECT/CT acquisitions were performed 1 hour after intravenous injection of 57.6 ± 9.8 MBq of ^99m^Tc-cAbVCAM1-5 or ^99m^Tc-Ctl. The cAbVCAM1-5 and control sdAbs were radiolabeled as previously described using the tricarbonyl method [20]. Whole body SPECT/CT acquisitions were performed using a dedicated system (nanoSPECT-CT; Mediso, Budapest, Hungary). For the experimental metastasis study, mice were injected with 61.9 ± 16.2 MBq of ^99m^Tc-cAbVCAM1-5 (*n* = 5) or the irrelevant ^99m^Tc-Ctl (*n* = 4). Biodistributions were also performed on healthy mice (*n* = 4). SPECT/CT acquisition was centered on the thoracic region. CT and SPECT acquisitions were reconstructed and fused using Nucline software (Mediso), and SPECT quantification based on CT was performed using VivoQuant^TM^ (InviCRO, Boston, MA, USA). For the xenograft tumor model, a 50 mm^3^ sphere was drawn at the center of the tumor on the basis of the CT image. For the metastasis assay, total quantification of pulmonary tracer uptake based on the CT image was performed. ^99m^Tc-sdAbs activity was expressed as a percentage of the injected dose per cm^3^ (% ID/cm^3^).

### 4.9. Post-Mortem Analysis

Two hours after injection and immediately following SPECT/CT image acquisitions, anesthetized mice were euthanized using CO_2_ and tumors (either subcutaneous or the whole lungs with metastasis) were harvested along with other organs. Samples were weighed and their radioactivity determined with a γ-counter (Wizard^2^, PerkinElmer, Courtaboeuf, France). Results were corrected for decay, injected dose and organ weight and expressed as % ID/g. Subcutaneous tumors were then immediately frozen in −40 °C isopentane, whereas metastatic lungs were inflated with a 1:1 mixture of PBS/Optimal Cutting Temperature prior being frozen. In order to investigate whether the VCAM-1 expression status determined in vitro on HCC70 and MDA-MB-231 cells was preserved in vivo, RT-qPCR was performed on HCC70 and MDA-MB-231 subcutaneous tumor slices and tissue samples. Moreover, due to the fact that ex vivo counting on lung with metastases reflects a mixture of healthy tissue and tumor uptake, lung autoradiography was performed to precisely evaluate the tumor uptake. To that purpose, 20 µm-thick lung slices, together with reference organs of known activities, were exposed overnight on an autoradiographic film which was then scanned using a phosphoimager (Fujifilm BAS-5000, FUJIFILM, Montigny, France). Slices were then stained with hematoxylin-eosin and regions of interest were delineated on tumor nodules, thereby allowing the quantification of ^99m^Tc-cAbVCAM1-5 or ^99m^Tc-Ctl uptake as a percentage of injected dose per gram (% ID/g).

### 4.10. Statistics

For the in vitro and mice experiments: Data were expressed as mean ± standard deviation and analyzed using an unpaired Mann Whitney test for inter group analysis. Significance of linear correlations was assessed with a Pearson’s test. *p* values < 0.05 were considered significant. Data were analyzed with Prism 7.0 (GraphPad Software).

For patients: The Student’s t-test was used to compare continuous variables. Overall survival (OS) was defined as the time between surgery and the date of death from any cause. The Kaplan-Meier method was used to produce survival curves and analyses of censored data were performed using Cox models. Data were analyzed with Prism 7.0 (GraphPad Software).

## 5. Conclusions

In the two mouse models of TNBC, SPECT imaging of VCAM-1 was successfully performed and the signal originated from the tumor was found to reflect hVCAM-1 expression by cancer cells rather than mVCAM-1 expression by the tumor stroma. ^99m^Tc-cAbVCAM1-5 can therefore be used as a preclinical tool to evaluate the role of VCAM-1 expression by tumor cells in tumor development and metastasis. In clinical practice, VCAM-1 expression has been reported to be correlated with poorer outcome in TNBC but also in other cancer type such as glioblastoma and ovarian cancer, and VCAM-1 imaging has been proposed as a tool for the assessment of tumor aggressiveness. Further studies will however be necessary to evaluate the prognostic value of ^99m^Tc-cAbVCAM1-5 tumor imaging in clinical practice.

## Supplementary Materials

The following are available online at https://www.mdpi.com/2072-6694/11/7/1039/s1, Figure S1: MDA-MB-231 cells overexpress VCAM-1 protein upon TNF-α. Table S1: Biodistribution of 99mTc-cAbVCAM1-5 in athymic nude mice bearing HCC70 and MDA-MB-231 xenografts. Figure S2: cAbVCAM1-5 binds to MDA-MB-231 but not to HCC70 cells. Figure S3: Affinity of 99mTc-cAbVCAM1-5 for mVCAM-1 and hVCAM-1. Figure S4: Representative Maximum Intensity Projection views of fused SPECT/CT images of HCC70 and MDA-MB-231 tumor-bearing mice 1h after i.v injection of 99mTc-cAbVCAM1-5 or 99mTc-Ctl. Figure S5: Correlation between tumor uptake as determined by SPECT image quantification and by ex vivo biodistribution studies. Figure S6: Representative Maximal Intensity (MIP) of fused SPECT/CT views of MDA-MB-231 lung metastases at 1h after i.v injection of 99mTc-cAbVCAM1-5 of 99mTc-Ctl. One lung-metastases free mouse wal also injected with 99mTc-cAbVCAM1-5. Figure S7: Ex vivo quantification of 99mTc-cAbVCAM1-5 in SCID mice bearing MDA-MB-231 lung metastasis.
