# Supplementary material for: In Vivo Assessment of VCAM-1 Expression by SPECT/CT Imaging in Mice Models of Human Triple Negative Breast Cancer
Source: Cancers (Basel). 2019 Jul 23;11(7):1039. doi: 10.3390/cancers11071039 (PMC6678795; doi:10.3390/cancers11071039)
Supplement: Supplementary file 1 [file cancers-11-01039-s001.pdf]

SUPPLEMENTARY DATA

RESULTS

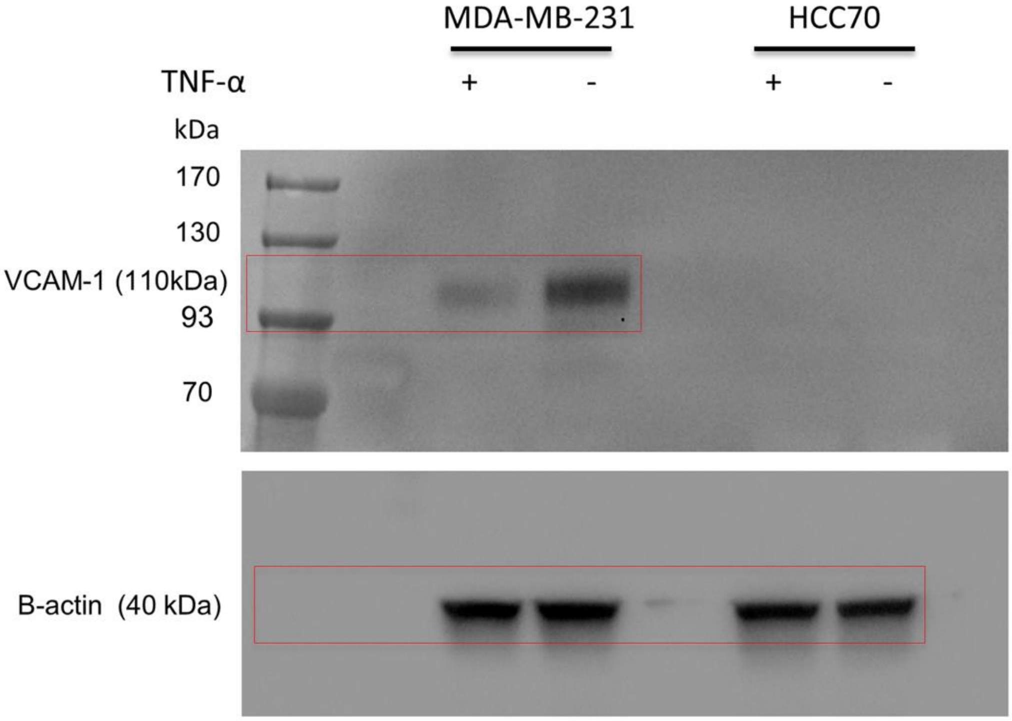

		MDA-MB-231	MDA-MB-231 + TNF-α
Band intensity (grey values)	VCAM-1	1- 7734<br>2- 12121<br>3- 60084	1- 40967<br>2- 44648<br>3- 190026
	Actin	1- 58771<br>2- 58296<br>3- 73757	1- 62827<br>2- 76114<br>3- 83096

Figure S1. MDA-MB-231 cells overexpress VCAM-1 protein upon TNF-*α* stimulation. MDA-MB-231 and HCC70 cells (200.000) were stimulated or not with TNF-*α* for 18 h and VCAM-1 expression was assessed by Western Blot. Grey values of 3 different experiments are indicated in the table.

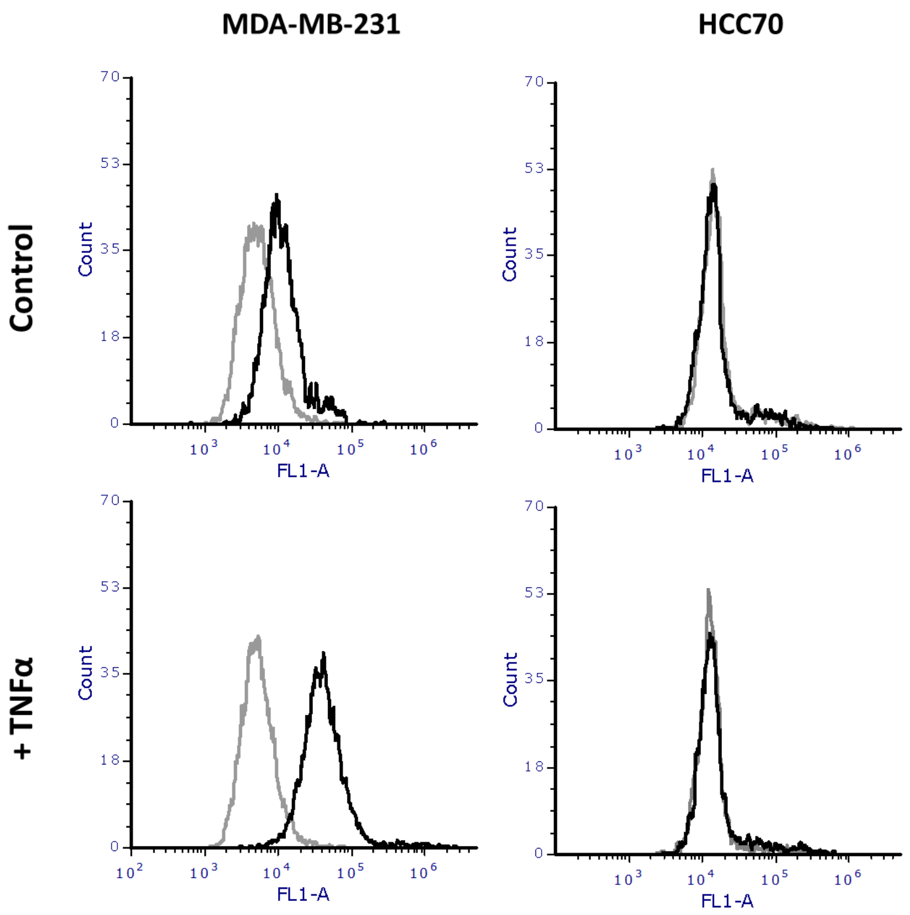

Figure S2. cAbVCAM1-5 binds to MDA-MB-231 but not to HCC70 cells. MDA-MB-231 and HCC70 cells (200.000) were stimulated with TNF-α for 18 h and cAbVCAM1-5 ability to bind to these cells was assessed by flow cytometry. Cells were then incubated with cAbVCAM1-5 (black line) or control sdAb (gray line) then with an anti-poly histidine antibody (1/200, anti-6x His tag® antibody, ab9108 Abcam), followed by a fluorochrome-conjugated monoclonal antibodies anti-Rabbit-IgG (1/500, goat anti-Rabbit IgG H&L, AlexaFluor® 488, ab150077 Abcam).

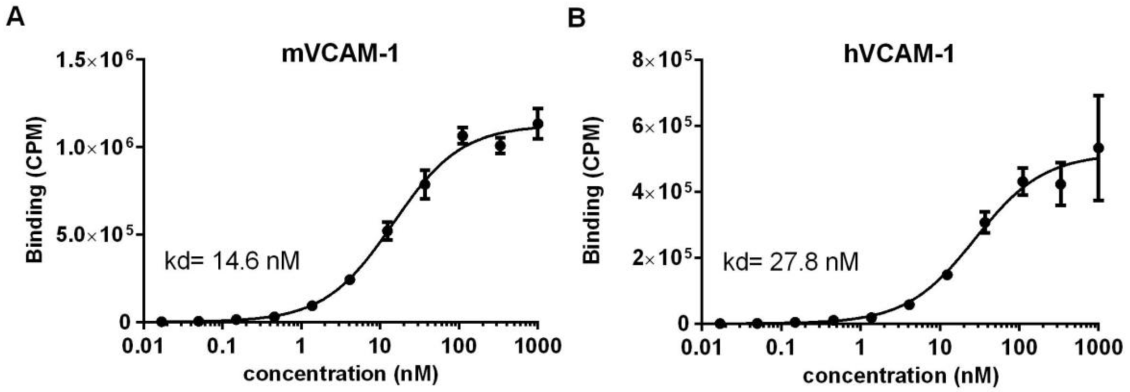

Figure S3. Affinity of ^{99m}Tc-cAbVCAM1-5 for mVCAM-1 and hVCAM-1. Saturation binding on (**A**) mouse VCAM-1 (mVCAM-1) and (**B**) human VCAM-1 (hVCAM-1).

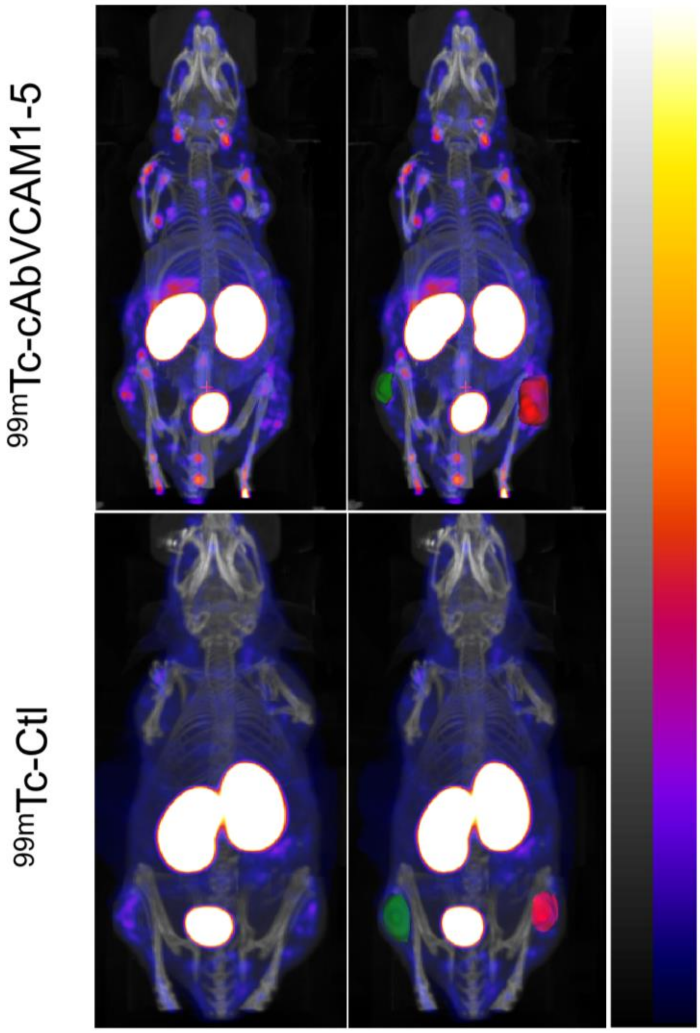

Figure S4. Representative Maximum Intensity Projection views of fused SPECT/CT images of HCC70 (right hind limb, red) and MDA-MB-231 (left hind limb, green) tumor-bearing mice at 1 h after i.v injection of ^{99m}Tc-cAbVCAM1-5 or ^{99m}Tc-Ctl.

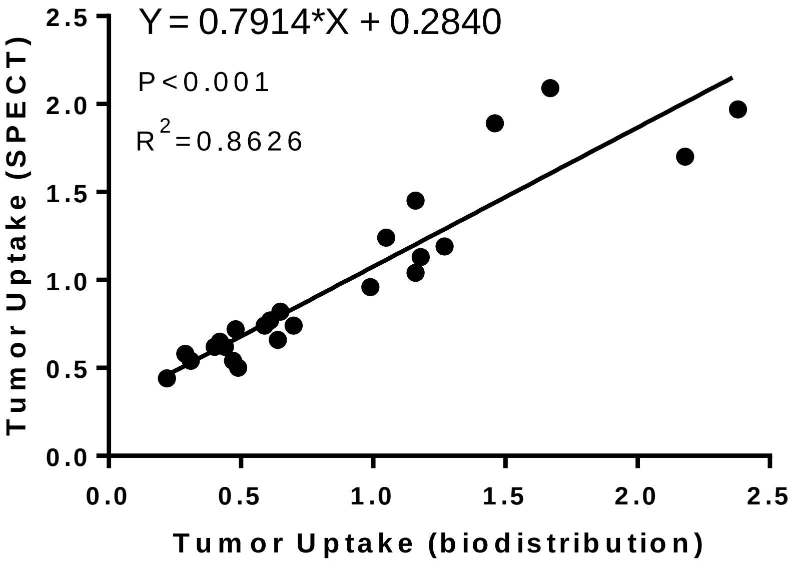

Figure S5. Correlation between tumor uptake as determined by SPECT image quantification and by ex vivo biodistribution studies.

Table 1. Biodistribution of ^{99m}Tc-cAbVCAM1-5 in athymic nude mice bearing HCC70 and MDA-MB-231 xenografts. Mice were euthanized 2 h after intravenous injection of ^{99m}Tc-cAbVCAM1-5 of ^{99m}Tc-Ctl. The organs were collected, weighed and radioactivity was measured by ◎-counter. Results were expressed as % ID/g or as tumor-to-blood ratio. * P < 0.05, ** P < 0.01 vs ^{99m}Tc-Ctl. ## P < 0.01 vs HCC70.

Organs	^{99m}Tc-cAbVCAM1-5	^{99m}Tc-Ctl
**Brain**	0.03 ± 0.02	0.01 ± 0.01
**Stomach**	0.49 ± 0.14	0.35 ± 0.10
**Intestin**	1.20 ± 0.48**	0.26 ± 0.06
**Liver**	0.73 ± 0.18**	0.44 ± 0.11
**Heart**	0.33 ± 0.10**	0.19 ± 0.07
**Kidney**	253.30 ± 33.87**	174.86 ± 32.51
**Lung**	1.88 ± 0.47**	0.52 ± 0.13
**Bone Marrow**	6.45 ± 2.38**	0.53 ± 0.24
**Spleen**	8.18 ± 1.19**	0.28 ± 0.20
**Lymph Nodes**	4.88 ± 0.49**	0.38 ± 0.17
**Blood**	0.37 ± 0.13	0.37 ± 0.11
**Muscle**	0.13 ± 0.07	0.10 ± 0.02
**MDA-MB-231**	1.69 ± 0.50**##	0.40 ± 0.08
**HCC70**	0.89 ± 0.27	0.60 ± 0.23
**MDA-MB-231/Blood**	5.14 ± 1.01**##	1.13 ± 0.23
**HCC70/Blood**	2.51 ± 0.68*	1.55 ± 0.39

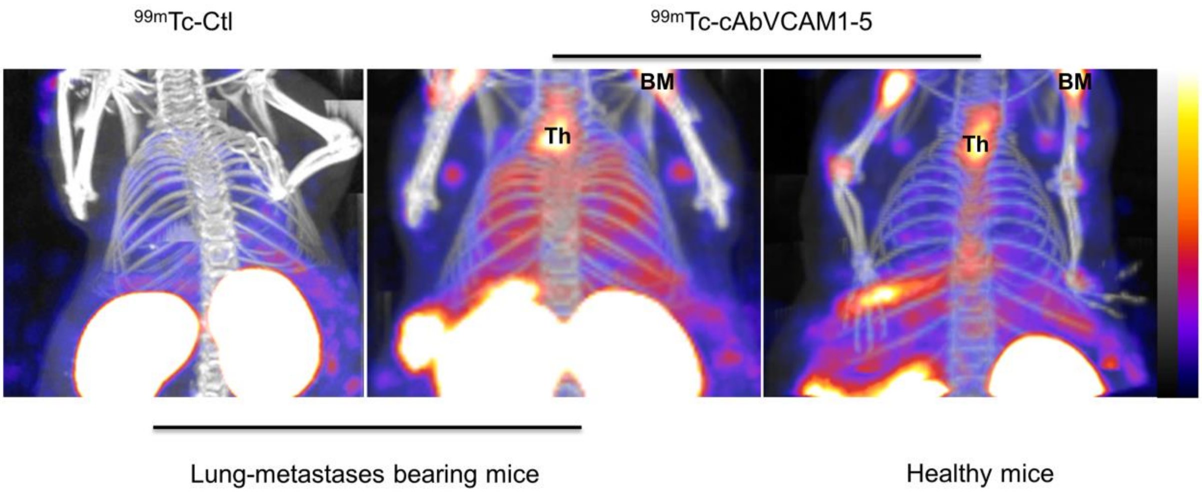

Figure S6. Representative Maximal Intensity Projection (MIP) of fused SPECT/CT views of MDA-MB-231 lung metastases at 1 h after i.v. injection of ^{99m}Tc-cAbVCAM1-5 (center) or ^{99m}Tc-Ctl (left). One lung-metastases free mouse was also injected with ^{99m}Tc-cAbVCAM1-5. BM: Bone Marrow, Th: Thymus.

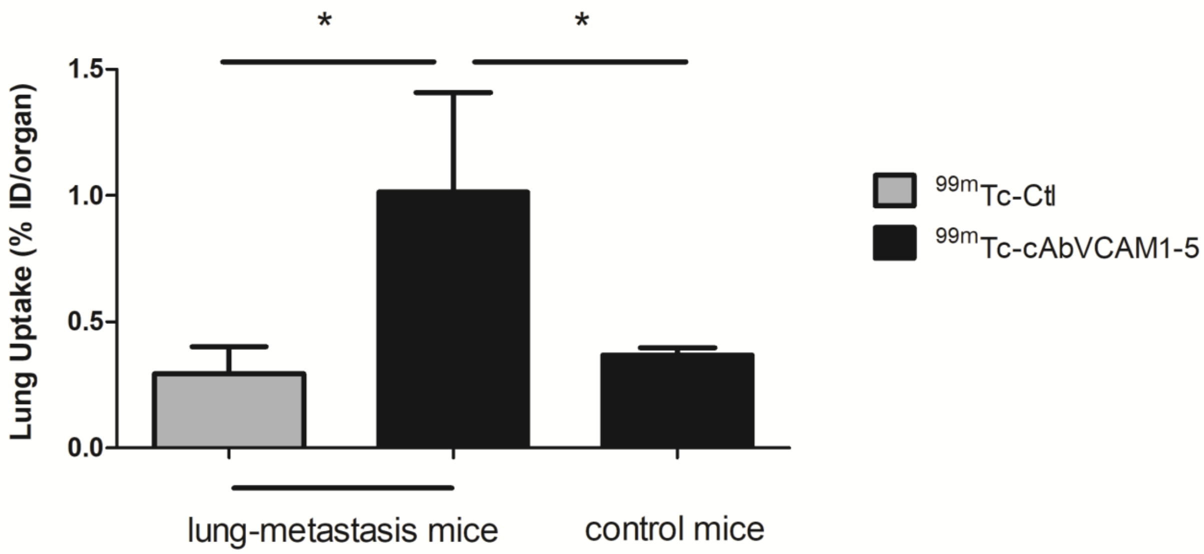

Figure S7. Ex vivo quantification of ^{99m}Tc-cAbVCAM1-5 in SCID mice bearing MDA-MB-231 lung metastasis. Lung-metastasis bearing or healthy mice were injected with ^{99m}Tc-cAbVCAM1-5 or ^{99m}Tc-Ctl and lung uptake was assessed 2 hours after injection of the radiotracers. Results were expressed in % ID/organ. Statistics * $P<0.05$.
